# Feeding State, Insulin and NPR-1 Modulate Chemoreceptor Gene Expression via Integration of Sensory and Circuit Inputs

**DOI:** 10.1371/journal.pgen.1004707

**Published:** 2014-10-30

**Authors:** Matthew Gruner, Dru Nelson, Ari Winbush, Rebecca Hintz, Leesun Ryu, Samuel H. Chung, Kyuhyung Kim, Chrisopher V. Gabel, Alexander M. van der Linden

**Affiliations:** 1 Department of Biology, University of Nevada, Reno, Nevada, United States of America; 2 Department of Agriculture, Nutrition and Veterinary Sciences, University of Nevada, Reno, Nevada, United States of America; 3 Department of Brain Science, Daegu Gyeongbuk Institute of Science and Technology (DGIST), Daegu, Korea; 4 Department of Physiology and Biophysics, Boston University School of Medicine, Boston, Massachusetts, United States of America; 5 Boston University Photonics Center, Boston, Massachusetts, United States of America; University of California San Francisco, United States of America

## Abstract

Feeding state and food availability can dramatically alter an animals' sensory response to chemicals in its environment. Dynamic changes in the expression of chemoreceptor genes may underlie some of these food and state-dependent changes in chemosensory behavior, but the mechanisms underlying these expression changes are unknown. Here, we identified a KIN-29 (SIK)-dependent chemoreceptor, *srh-234*, in *C. elegans* whose expression in the ADL sensory neuron type is regulated by integration of sensory and internal feeding state signals. We show that in addition to KIN-29, signaling is mediated by the DAF-2 insulin-like receptor, OCR-2 TRPV channel, and NPR-1 neuropeptide receptor. Cell-specific rescue experiments suggest that DAF-2 and OCR-2 act in ADL, while NPR-1 acts in the RMG interneurons. NPR-1-mediated regulation of *srh-234* is dependent on gap-junctions, implying that circuit inputs regulate the expression of chemoreceptor genes in sensory neurons. Using physical and genetic manipulation of ADL neurons, we show that sensory inputs from food presence and ADL neural output regulate *srh-234* expression. While KIN-29 and DAF-2 act primarily via the MEF-2 (MEF2) and DAF-16 (FOXO) transcription factors to regulate *srh-234* expression in ADL neurons, OCR-2 and NPR-1 likely act via a calcium-dependent but MEF-2- and DAF-16-independent pathway. Together, our results suggest that sensory- and circuit-mediated regulation of chemoreceptor genes via multiple pathways may allow animals to precisely regulate and fine-tune their chemosensory responses as a function of internal and external conditions.

## Author Summary

Animals dramatically modify their chemosensory behaviors to attractive and noxious chemical stimuli when starved. This could allow them to alter and optimize their food-search strategies to increase their survival and reproduction. Changes in the gene expression of chemoreceptors specialized in detecting environmental stimuli is observed in fish, insects and nematodes, and may be a general mechanism underlying the changes in chemosensory behaviors observed in starved animals. To elucidate this mechanism, we have developed an *in vivo* reporter assay in *C. elegans* for monitoring the expression of a candidate chemoreceptor gene in a single sensory neuron type, called ADL, as a function of feeding state. Using this reporter assay, we show that sensory inputs into ADL and neural outputs from ADL, as well as inputs from the RMG interneuron, which is electrically connected to ADL, are required to fine-tune expression of chemoreceptor genes in ADL. Sensory and circuit-mediated regulation of chemoreceptor gene expression is dependent on multiple pathways, including the neuropeptide receptor, NPR-1, and the DAF-2 insulin-like receptor. Our results reveal mechanisms underlying chemoreceptor gene expression, and provide insight into how expression changes in chemoreceptor genes may contribute to changes in chemosensory behavior as a function of feeding state.

## Introduction

An animals' feeding state (i.e. fed *versus* starved) and food availability dramatically alters the responsiveness of chemosensory neurons and behavioral output to suit the needs of the animal to, for instance, locate food, find mates and avoid predators under different environmental conditions. Although these state-dependent changes in chemosensory behaviors have long been thought to arise from plasticity in central processes, there is now growing evidence that feeding state gates responses in peripheral chemosensory neurons themselves, thereby directly modulating changes in chemosensory behaviors [1]. One particular mechanism by which feeding state could alter the response properties of chemosensory neurons, is by dynamically changing the transcript levels of chemoreceptor genes. For example, in mosquitoes, olfactory neuron responsiveness to host-specific odors after a blood-feeding is highly correlated with small changes in transcript levels of the corresponding olfactory receptor [2]–[5], which may allow them to alter their sensitivity to these odors, thereby altering its host-seeking behavior. Thus, dynamic changes in the expression levels of chemoreceptor genes may provide a simple mechanism by which chemosensory neurons may alter their responses to specific chemical stimuli under different feeding state conditions.

The *Caenorhabditis elegans* sensory system provides an ideal system to explore the mechanisms by which chemosensory gene expression and behavior is altered by changes in its environment. As in other animals, *C. elegans* is able to rapidly and reversibly modify its chemosensory behavior to specific chemicals according to its feeding state. For example, starved animals increase their adaptation towards particular attractive odors and they discriminate more classes of food-related odors than fed animals [6]. Starved animals also change their response to certain volatile repulsive odors, and this modulation occurs through serotonergic and dopaminergic signaling [7], [8]. The neuropeptide receptor, NPR-1, and insulin-like receptor, DAF-2, in *C. elegans* are also involved in a multitude of food-dependent behaviors. For example, pre-exposure of *C. elegans* to salt in the absence of food switches the normally attractive salt response to avoidance, and this modulation is dependent on insulin signaling from the AIA interneurons via DAF-2 acting in ASE sensory neurons [9], [10]. Recent elegant work has shown that the neuropeptide receptor NPR-1 plays a key role in the RMG interneuron to regulate avoidance responses of sensory neurons to pheromones, a response that appears to be mediated by food [11], [12]. Thus, insulin and neuropeptide signaling from interneurons play an important role in translating multiple aspects of food and feeding state information to peripheral chemosensory neurons to fine-tune their responses.

As described above, dynamic changes in the expression levels of chemoreceptor genes could, at least in part, contribute to modifications in chemosensory behaviors, but it is unknown whether or how neuromodulators, such as neuropeptides, insulin and monoamines, alter expression levels of chemoreceptor genes as a function of feeding state. In *C. elegans*, individual and distinct subsets of chemoreceptor genes are regulated by several mechanisms, including developmental changes, sensory activity, and levels of pheromones [13],[14]. In addition, our previous work showed that KIN-29 regulates a subset of chemoreceptors in chemosensory neurons [15] by phosphorylating the HDA-4 class II histone deacetylase (HDAC), and inhibiting the gene repressive functions of the MEF-2 transcription factor [16]. KIN-29 is a member of the salt-inducible kinase (SIK) family, which plays a major role in the regulation of lipolysis and gluconeogenic gene expression in response to feeding and fasting [17]–[20]. For example, during feeding, *Drosophila* SIK3 is activated by insulin to regulate fat stores through phosphorylation of HDAC4 [21], a function that appears to be conserved in *C. elegans* [16]. Upon starvation, *Drosophila* SIK3 is inactivated resulting in HDAC4 dephosphorylation and subsequent activation of FOXO-mediated gene expression [21]. Thus, SIKs are critical regulators of feeding and fasting states, suggesting that a role for KIN-29 in feeding-state dependent regulation of gene expression may be conserved.

Here, we identified a KIN-29-dependent chemoreceptor, *srh-234*, whose expression levels in the ADL sensory neuron type of *C. elegans* is downregulated upon starvation. We find that this starvation-modulation is likely a consequence of both sensory inputs associated with a decrease in food presence and an internal state of starvation due to a decrease in food ingestion. We show that in addition to KIN-29, expression levels of *srh-234* are regulated by multiple pathways, including signaling mediated by the DAF-2 insulin-like receptor, OCR-2 TRPV channel, and NPR-1 neuropeptide receptor. We show that intact cilia and dendrites of ADL, as well as neural output from ADL are required for *srh-234* expression. Cell- and tissue-specific rescue experiments show that DAF-2 and OCR-2 act in ADL neurons, whereas NPR-1 acts in RMG interneurons to regulate *srh-234* expression and this regulation is dependent on *unc-7/9* gap-junctions. While MEF-2 and DAF-16 FOXO transcription factors act downstream of KIN-29 and DAF-2, respectively, in regulating *srh-234* expression, OCR-2 and NPR-1 pathways act independently from MEF-2 and DAF-16. Taken together, our results suggest that integration of sensory and circuit inputs via multiple signaling pathways allows animals to precisely modulate chemoreceptors genes according to their feeding status, providing insights into the gene expression mechanisms that contribute to chemosensory plasticity in *C. elegans*.

## Results

### Expression of *srh-234* in ADL neurons is downregulated in starved animals

**Figure 1:**
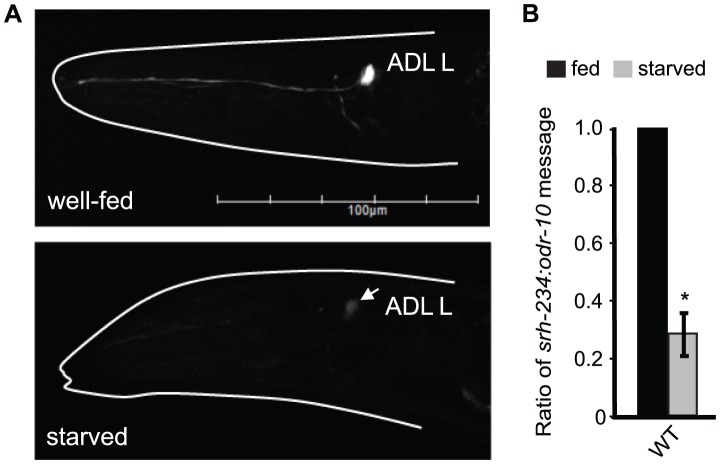
Starvation downregulates the expression of *srh-234* in ADL neurons. A) Expression of *srh-234*p::*gfp* in an ADL neuron of wild-type animals when well-fed in the presence of *E. coli* OP50 food (upper panel) and starved (lower panel) in the absence of food for 12 hours. The head of the animal is indicated with a white line. Arrow points to the ADL cell body. Images were acquired at the same exposure time. Lateral view: anterior is on the left. Scale, 100 µm. B) Levels of endogenous *srh-234* messages are downregulated in starved animals compared to fed animals. Shown is the ratio of endogenous *srh-234* message to endogenous *odr-10* [65] message as quantified by qRT–PCR in fed and starved animals. In hermaphrodite animals, expression of *odr-10* is unaffected by starvation. The mean of the ratios from two independent experiments is shown. * indicates values that are different from fed wild-type animals at *P*<0.01 using a two-sample *t*-test. Error bars denote the SEM.

Since SIKs regulate feeding state-dependent gene expression, we investigated whether feeding and starvation regulates the expression of *kin-29*-dependent chemoreceptor genes. Expression of *gfp* driven under the regulatory sequences of candidate chemoreceptor genes, *str-1*, *sra-6* and *srh-234* is strongly downregulated in AWB, ASH and ADL neurons, respectively [15], [16]. We found that gfp expression driven under only 165 bp of the regulatory sequence of *srh-234* (*srh-234p::gfp*) is strongly expressed in ADL in fed animals (Figure 1A, upper panel), but when animals were starved for long-periods of time (>6 hours), *srh-234p::gfp* was significantly downregulated (Figure 1A, lower panel). Similar results were found for another independent integrated transgenic array of *srh-234p::gfp* (*oyIs57*) (Table S2). The expression of *str-1::gfp* and *sra-6::gfp* was unaffected in starved animals. We further confirmed the starvation-induced change in expression by examining the endogenous levels of *srh-234* with help of qRT-PCRs, and found that the transcript levels of *srh-234* were similarly downregulated but not abolished in starved animals (Figure 1B). The effect of starvation on *srh-234* expression is reversible as L1 larvae or adult animals starved for 12 hours and then re-fed with *E. coli* food restore expression to near wild-type levels within 6 hours (Figure S1A). Moreover, when starved L1 larvae were grown on a nutrient-rich minimal media for 24 hours that is axenic, i.e. in the absence of any bacterial food, allowing developmental growth albeit delayed {Szewczyk, 2003 #4559], no increase in *srh-234* expression was observed (Figure S1B). As bacterial food can alter the production of pheromones in *C. elegans* [22], which in turn can regulate chemoreceptor gene expression [13], we examined *srh-234* expression in the absence and presence of pheromones. However, no effect on *srh-234* expression was found in *daf-22(m130)* mutants, which is required for pheromone biosynthesis [23], and in the presence of crude pheromone extracts (Table S2), suggesting that pheromones do not alter *srh-234* expression. Thus, starvation reduces the expression of the *kin-29*-dependent chemoreceptor, *srh-234*, in ADL, and this modulation is reversible upon re-feeding with food.

We next sought to identify additional chemoreceptor genes in ADL regulated by fed and starved conditions. Of the 11 chemoreceptor genes tested, only *gfp* driven by the *cis*-regulatory region of the chemoreceptor *srh-34* in ADL was altered in fed and starved animals with an opposite phenotype than observed for *srh-234*; *srh-34* is expressed in starved animals but not in fed animals (Table S1). Thus, the feeding status (fed *versus* starved) of *C. elegans* alters the expression levels of at least two chemoreceptor genes in ADL. We focused our subsequent studies on using the *srh-234*p::*gfp* reporter assay to further explore the mechanisms underlying starvation modulation of chemoreceptor gene expression in ADL.

### External food presence and an internal state of starvation modulate *srh-234* expression

**Figure 2:**
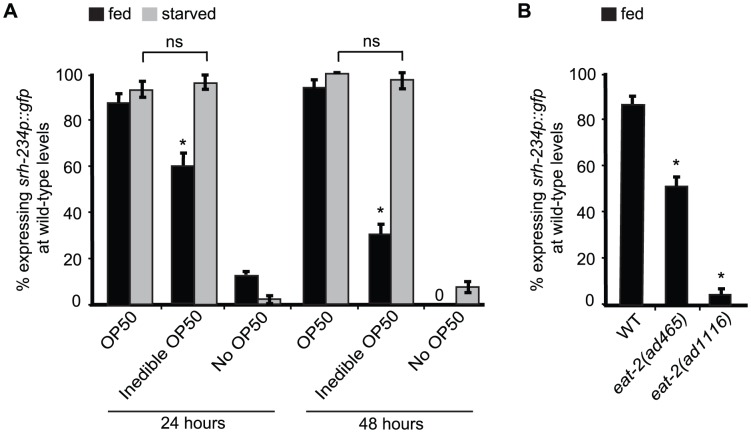
External food presence and internal state signals alter *srh-234* expression levels. A) Percentage of animals expressing *srh-234*p::*gfp* at wild-type levels when fed with *E. coli* OP50 food (OP50), aztreonam-treated *E. coli* OP50 food (inedible OP50), or no food (no OP50) for 24 and 48 hours. Young adult animals grown on edible *E. coli* OP50 food were divided into two groups: a fed group maintained in the presence of food, and a starved group maintained in the absence of food for 6–12 hours. We confirmed that *srh-234* expression levels upon starvation were reduced. Subsequently, adults were picked onto new NGM plates seeded with either edible *E. coli* OP50, no *E. coli* OP50, or inedible *E. coli* OP50 food (see Material and Methods). B) Percentage of *eat-2(lf)* mutants defective in food intake expressing *srh-234*p::*gfp* at wild-type levels. In all experiments, wild-type expression of *srh-234*p::*gfp* was defined as expression levels that allowed visualization of both the cell bodies and processes of at least one ADL neuron (see Material and Methods). Animals (n>150) were examined at 150× magnification for each condition or genotype. * indicates values that is different from that of wild-type animals at *P*<0.001, and n.s. indicates the values that are not significantly different between the different food conditions compared by brackets using a χ^2^ test of independence. Error bars denote the SEP.

The reduced *srh-234* expression levels we observed in starved wild-type animals could arise as a consequence of an internal state of starvation triggered by a decrease in the ingestion of food, or alternatively, by an external sensory response as a result of a decrease in the perception of food. To distinguish between these possibilities, we exposed fed and starved wild-type adult animals carrying the *srh-234*p::*gfp* reporter to *E. coli* food treated with the antibiotic aztreonam, which results in bacteria that grow in long chains that *C. elegans* cannot eat due its large size, but still can smell and touch comparable to regular non-treated bacteria [24]. We found that fed animals placed on aztreonam-treated *E. coli* OP50 (inedible food) for 24 and 48 hours significantly reduces *srh-234* expression mimicking the effects of starvation when compared to fed animals placed on non-treated *E. coli* OP50 (edible food) (Figure 2A). Consistent with these findings, loss-of-function (*lf*) mutations in *eat-2* that result in animals with a pharyngeal pumping defect compromising their food ingestion, also reduce *srh-234* expression on edible food (Figure 2B). Thus, the reduced *srh-234* expression is likely due to an internal state response triggered by a decreased food ingestion. However, when we placed starved adult animals (in the absence of food for 6–12 hours) on aztreonam-treated *E. coli* OP50 (inedible food), we found that the *srh-234* expression phenotype was not significantly different from their non-treated *E. coli* OP50 (edible food) counterpart as if they sense food presence correctly even when they cannot eat this food (Figure 2A). It is possible that starved animals in our experiments can ingest some of the inedible food but at a reduced amount. However, we found that animals placed on aztreonam-treated *E. coli* food have a starved appearance similar to starved animals placed on plates without any food. Moreover, we verified that aztreonam-treated *E. coli* cannot be eaten properly as we find that L1 larvae exposed to treated food used in our experiments do not sustain growth (98% of L1 animals placed on aztreonam-treated bacteria for 24 hours were arrested, as compared to 0% of L1 larvae placed on edible food). Thus, the perception of inedible food can override the effects of starvation on reducing *srh-234* expression levels. In summary, our results suggest that the starvation-induced downregulation of *srh-234* expression is likely a consequence of both sensory inputs associated with a decreased food presence, and an internal state of starvation triggered by a decrease in food ingestion.

### DAF-2/DAF-16 acts cell-autonomously in ADL to regulate *srh-234* expression

**Figure 3:**
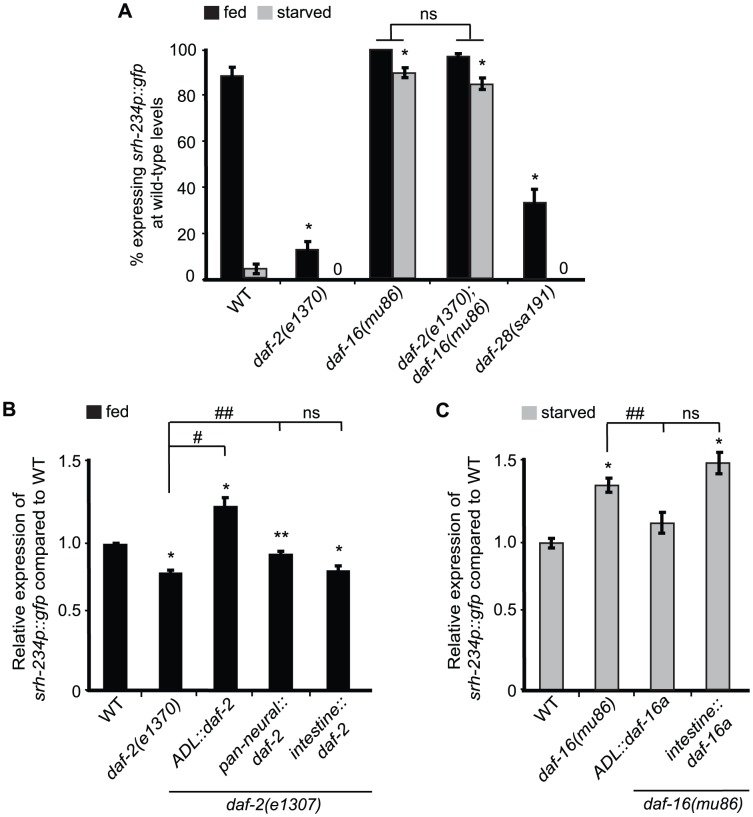
*daf-2* and *daf-16a* are required in ADL, but not in the intestine, to regulate *srh-234* expression. A) Percentage of mutant animals with defects in insulin signaling expressing *srh-234*p::*gfp* at wild-type levels. *daf-2(e1307)* is a temperature sensitive allele. In all experiments, animals were raised at 15°C (permissive temperature) and shifted to the 25°C (restrictive temperature) as L4 larvae. Animals (n>150) were examined at 150× magnification for each genotype. B, C) Relative expression of *srh-234*p::*gfp* in *daf-2* or *daf-16* mutants carrying compared to wild-type when fed or starved. For strains carrying *ADL::daf-2* cDNA, *pan-neural::daf-2* cDNA, *intestine::daf-2* cDNA, *ADL::daf-16a* cDNA and *intestine::daf-16a* cDNA extrachromosomal arrays (see Material and Methods), data shown is for at least two independent transgenic lines. Animals (n = 18–25) were examined at 400× magnification for each genotype. * indicates values that are different from that of wild-type animals at *P*<0.001, # and ## indicates the values that are different between the genotypes compared by brackets at *P*<0.001, and *P*<0.05, respectively, using either a two-sample *t*-test or a χ^2^ test of independence. Error bars denote the SEM or SEP.

As internal state signals in *C. elegans* are conveyed through an insulin signaling pathway with DAF-2 being the main insulin-like receptor [25], we explored whether insulin signaling plays a role in the regulation of *srh-234*. Consistent with low *daf-2* insulin signaling being associated with a starved state, we found that *daf-2(e1307)* mutants reduce *srh-234* expression in ADL in fed conditions, similar to starved wild-type animals (Figure 3A). Since *daf-2* activates insulin signaling by repressing the *daf-16* FOXO transcription factor, and loss of *daf-16* function results in active insulin signaling [26], we next examined whether *daf-16(mu86)* suppressed the *daf-2*- and starvation-induced downregulation of *srh-234* expression. Indeed, we found that both *daf-16(mu86)* mutants and *daf-2(e1307); daf-16(mu86)* double mutants showed a significant increase in *srh-234* expression during starvation compared to starved wild-type animals (Figure 3A), suggesting that starved animals reduce *srh-234* expression by lowering DAF-2 signaling and activating DAF-16.

To further refine the site of action of the DAF-2/DAF-16 insulin signaling pathway in regulating *srh-234* expression, we introduced cDNAs of *daf-2* and *daf-16* under different cell- and tissue-specific promoters in *daf-2* and *daf-16* mutants, respectively, and measured their effects on *srh-234* expression in either fed or starved conditions. We found that expression of *daf-2* in ADL neurons fully restored the reduced *srh-234* expression phenotype of *daf-2(e1307)* mutants during feeding to near wild-type levels, whereas *daf-2* expression in the nervous system had a partial effect (Figure 3B). Expression of *daf-2* in the intestine did not result in a restoration of the reduced *srh-234* expression phenotype of *daf-2(e1307)* mutants (Figure 3B). These results suggest that DAF-2 signaling acts in ADL to regulate *srh-234* expression. There are three functionally characterized DAF-16 isoforms, DAF-16a, DAF-16b and DAF-16df, and all of these isoforms show neuronal expression in developing larvae [27]–[29]. We found that expression of *daf-16a* cDNA specifically in ADL restored the increased *srh-234* expression of *daf-16(mu86)* mutants during starvation back to wild-type levels, but similar to *daf-2*, expression of *daf-16a* cDNA in the intestine had no effect (Figure 3C). Together, these results suggest that both DAF-2 and DAF-16 act cell-autonomously in ADL to regulate *srh-234* expression levels.

There are over 40 insulin-like peptides (ILPs) expressed in *C. elegans*. The *daf-28* ILP, is a known agonist for DAF-2 and is expressed at high levels only when food is present [30],[31]. Interestingly, a semi-dominant mutation, *sa191*, in *daf-28*, thought to block other agonistic ILPs through stereo-hindrance [32], partially reduces *srh-234* expression in ADL during feeding (Figure 3A). These results suggest that DAF-28 or other ILPs regulate *srh-234* expression in ADL, likely through the DAF-2/DAF-16 insulin pathway.

### NPR-1 acts in RMG to regulate *srh-234* expression

**Figure 4:**
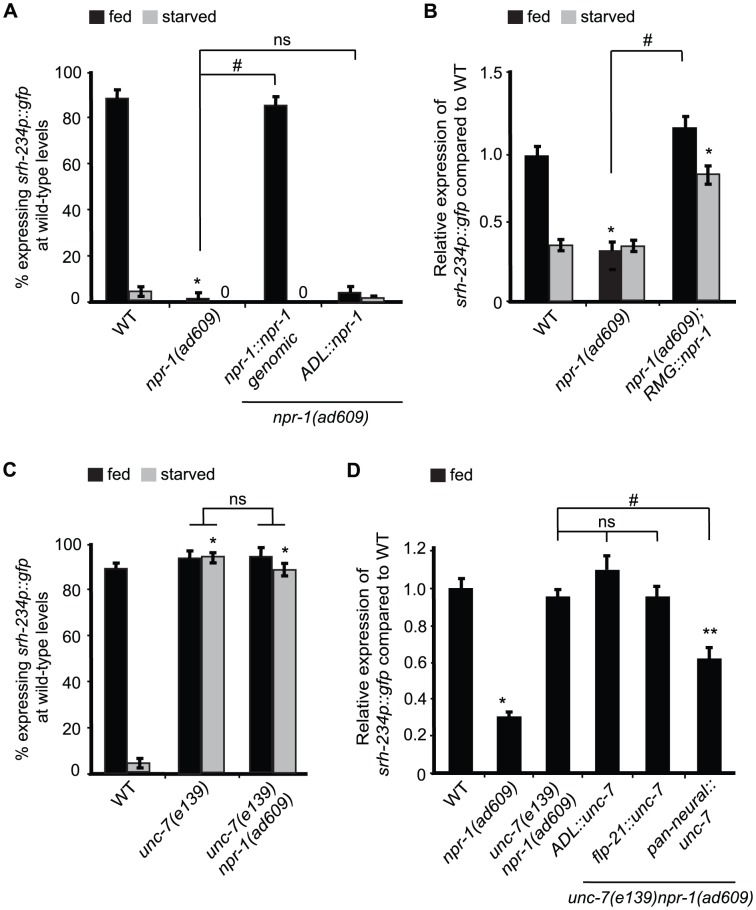
Reducing *npr-1* activity in RMG promotes *srh-234* expression levels. A) Percentage of *npr-1* mutant animals expressing *srh-234*p::*gfp* at wild-type levels. For strains carrying the *npr-1::npr-1* genomic and *ADL::npr-1* extrachromosomal arrays (see Material and Methods), data shown is the average of at least two independent transgenic lines. Animals (n>150) were examined at 150× magnification for each genotype. B) Relative expression of *srh-234*p::*gfp* in *npr-1* mutants compared to wild-type. For strains carrying *RMG::npr-1* extrachromosomal arrays (see Material and Methods), data shown is for at least two independent transgenic lines. Animals (n = 20–23) were examined at 400× magnification for each genotype. C) Percentage of animals of the indicated genotypes expressing *srh-234*p::*gfp* at wild-type levels. Animals (n>150) were examined at 150× magnification for each genotype. D) Relative expression of *srh-234*p::*gfp* in *unc-7 npr-1* double mutants compared to wild-type. For strains carrying *ADL::unc-7L cDNA*, *flp-21::unc-7L* cDNA and *pan-neural::unc-7L* cDNA extrachromosomal arrays (see Material and Methods), data shown is for at least two independent transgenic lines. Animals (n = 15–22) were examined at 400× magnification for each genotype. In all experiments, * indicates values that is different from that of wild-type animals at *P*<0.001, and # indicates the values that are different between the genotypes compared by brackets at *P*<0.001 using either a χ^2^ test of independence or using a two-sample *t*-test. n.s. indicates the values between brackets that are not significantly different. Error bars denote the SEM or SEP.

The neuropeptide receptor, NPR-1, in *C. elegans* regulates a range of food-related behaviors. For example, mutants lacking *npr-1* move rapidly, avoid high oxygen concentrations and aggregate in groups in a food-dependent manner [33]–[35]. We therefore examined whether loss of NPR-1 activity alters the expression levels of *srh-234* in ADL. Indeed, we found a strong reduction in *srh-234* expression in ADL in *lf* mutants of *npr-1* (alleles *ad609*, *ky13* and *ok1447*), and in a reduction-of-function *npr-1* allele, *g320*, in fed conditions, with the *g320* allele having the weakest effect (Figure 4A; Table S2). *lf* mutations in *flp-18* and *flp-21* encoding NPR-1 ligands as well as double mutants inactivating both ligands did not alter *srh-234* expression (Table S2), suggesting that neuropeptides other than FLP-18/FLP-21 may act on NPR-1 to regulate *srh-234* expression. Expression of *npr-1* under control of its own promoter fully restored the reduced *srh-234* expression phenotype of *npr-1(ad609)* mutants back to wild-type levels (Figure 4A). However, ADL-specific expression of *npr-1* using the *sre-1* promoter did not restore the reduced *srh-234* expression phenotype of *npr-1(ad609)* mutants (Figure 4A), suggesting that *npr-1* activity is required in neurons other than ADL to regulate *srh-234* expression.

We next sought to identify the cells where *npr-1* activity is required for regulation of *srh-234* expression in ADL. *npr-1* is expressed in at least 20 cells [36]; 4 of these form chemical synapses with ADL, and 3 form gap-junctions with ADL, including RMG interneurons [37]. Previous studies have shown that RMG is the major site for NPR-1 in regulating aggregative behavior and pheromone responses [11], [12]. Based on these findings, we asked whether similarly RMG is the site of action for NPR-1 to modulate *srh-234* expression in ADL. Cell-specific expression of *npr-1* in RMG using a previously described Cre-Lox system (referred further as *RMG::npr-1*) [11] in *npr-1(ad609)* mutants completely restored the reduced *srh-234* expression phenotype of *npr-1* mutants to wild-type levels during feeding, and increased *srh-234* expression when starved (Figure 4B). This increase is likely due to overexpression effects of the *RMG::npr-1* transgene, which could overwhelm inhibitory regulation of *srh-234* by starvation. Thus, *npr-1* is necessary and sufficient in RMG to promote *srh-234* expression.

We next asked how NPR-1 in RMG interneurons affects *srh-234* expression in ADL? Reasoning by analogy to the recently proposed RMG hub-and-spoke circuit [11] we examined whether loss of gap-junction function alters *srh-234* expression in ADL. The innexins, *unc-7* and *unc-9*, are widely expressed in neurons and muscles and form electrical synapses in the locomotory system [38], and *unc-9* is involved in the modulation of ADL-mediated pheromone responses [12]. We found that *unc-7(e139)* and *unc-9(e101)* fully suppressed the reduced *srh-234* expression phenotype of *npr-1(ad609)* mutants in fed conditions (Figure 4C), but no suppression was observed in a *daf-2(e1307)* or *kin-29(oy38)* mutant background (Table S2). These results suggest that *unc-7/9* gap junctions are necessary for *npr-1*-mediated regulation of *srh-234* expression; however, other signaling pathways such as *ocr-2* and *daf-2* may act in parallel on the *srh-234* promoter. We also observed that *srh-234* expression in starved conditions is significantly upregulated in *unc-7(e139)* and *unc-9(e101)* mutants, as well as in *unc-7 npr-1* and *unc-9 npr-1* double mutants when compared to starved wild-type animals (Figure 4C; Table S2), suggesting that loss of *unc-7/9* gap-junctions can suppress the effects of starvation on reducing *srh-234* expression.

We next investigated whether UNC-7/9 are directly involved in the RMG gap-junction circuit to regulate *srh-234* expression. We therefore expressed the cDNA of the *unc*-7 specifically in ADL neurons (*ADL::unc-7*), in *flp-21*-expressing cells that include RMG interneurons (*flp-21::unc-7*), and in all neurons (*pan-neural::unc-7*) in *unc-7(e139) npr-1(ad609)* double mutants, and examined their effect on *srh-234* expression. Surprisingly, however, we did not observe a restoration of the reduced *srh-234* expression phenotype of *unc-7 npr-1* double mutants back to *npr-1* levels for either transgene in fed conditions (Figure 4D). Moreover, ADL-specific knock down of *unc-7* or *unc-9* by RNAi did not suppress the reduced *srh-234* expression phenotype of *npr-1(ad609)* mutants in fed conditions (Figure S3). Only pan-neural expression of *unc-7* cDNA showed a partial suppression of the reduced *srh-234* expression phenotype of *unc-7 npr-1* double mutants (Figure 4D). Thus, UNC-7/9 may have indirect effects on the regulation of *srh-234* expression mediated by NPR-1, although they could have subtle roles within the RMG gap-junction circuit.

### Sensory inputs into ADL and neural outputs from ADL regulate *srh-234* expression levels

**Figure 5:**
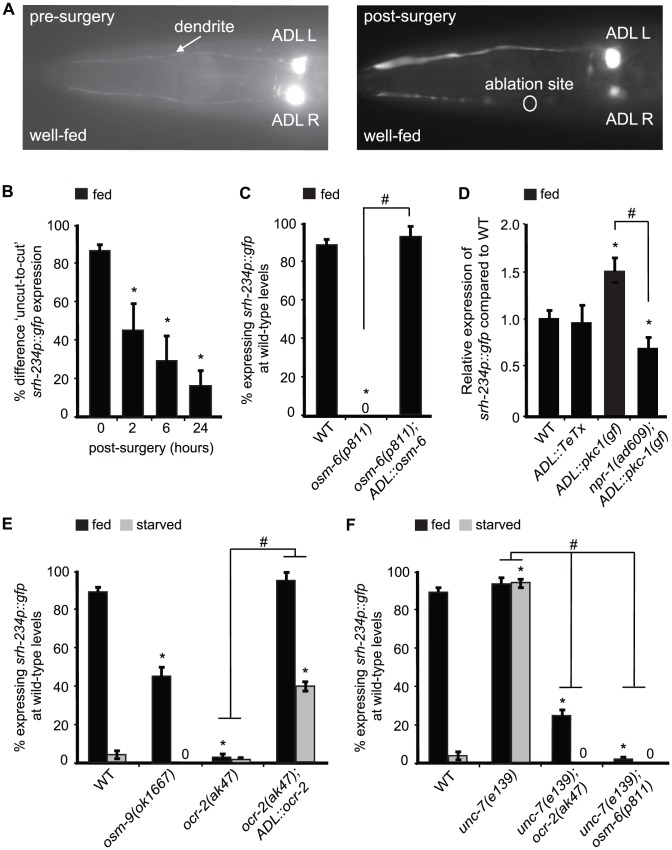
Expression levels of *srh-234* are modulated by sensory inputs into ADL, neural outputs from ADL and OCR-2 activity. A) Pictures show examples of fed animals expressing *srh-234*p::*gfp* before (left panel) and after surgery (right panel; 24 hr) of the right ADL (ADLR) dendrite using a femtosecond laser (see Material and Methods). The ablation site is indicated as a circle. B) Difference in fed animals expressing *srh-234*p::*gfp* between severed and non-severed (‘cut-to-uncut’) ADL sensory dendrites. Animals (n = 8–17) were examined at 400× magnification for each genotype. C) Percentage of mutant animals with defects in cilia formation expressing *srh-234*p::*gfp* at wild-type levels. For strains carrying the *ADL::osm-6* extrachromosomal array (see Material and Methods), data shown is the average of two independent transgenic lines. Animals (n>150) were examined at 150× magnification. D) Relative expression of *srh-234*p::*gfp* in the indicated genotypes compared to wild-type animals. For strains carrying *ADL::pkc-1(gf)* or *ADL::TeTx* extrachromosomal arrays, data shown is for two independent transgenic lines. Animals (n = 10–17) were examined at 400× magnification for each genotype. E, F) Percentage of animals of the indicated genotypes expressing *srh-234*p::*gfp* at wild-type levels. For strains carrying the *ADL::ocr-2* extrachromosomal array (see Material and Methods), data shown is the average of two independent transgenic lines. Animals (n>150) were examined at 150× magnification. * indicates values that are different from that of wild-type animals at *P*<0.001, and # between the genotypes compared by brackets at *P*<0.001 using either a two-sample *t*-test, or the using a χ^2^ test of independence. Error bars denote the SEP or SEM.

In addition to inputs from RMG facilitated by NPR-1, our experiments with aztreonam-treated *E. coli* food that can be sensed but not eaten suggest that *srh-234* expression is also modulated by sensory inputs from food. The presence of food could be perceived directly by ADL, or through other sensory neurons that connect to ADL. To examine these possibilities, we first performed physical and genetic manipulations of ADL sensory dendrites, thereby eliminating the ability of ADL neurons to perceive any sensory inputs from the environment. We analyzed *srh-234* expression in wild-type animals in which dendrites of the bilateral ADL pair are physically cut with a femtosecond laser (Figure 5A). This subcellular laser surgery exhibits precision with sub-micrometer resolution and has been successfully used in *C. elegans* to analyze the role of AFD sensory dendrites in temperature sensation [39]. We found that cutting either the ADLL or ADLR sensory dendrite showed a significant reduction in *srh-234* expression over time (Figure 5B). This is in contrast to AWB neurons where expression of *str-1p::gfp* is maintained after severing sensory dendrites [40]. Thus, ADL dendrites are necessary to promote *srh-234* expression in fed conditions.

Although these dendritic cuts indicate a direct role for ADL sensory neurons in regulating *srh-234* expression, it is possible that these subcellular cuts could damage ADL and thus compromise their physiology. To further confirm whether sensory inputs into ADL regulate *srh-234* expression, we examined *osm-5(p813)* and *osm-6(p811)* mutants that lack functional sensory cilia [41], [42], and found that these cilia defective mutants strongly reduce *srh-234* expression in ADL in fed conditions (Figure 5C). Functional reconstitution of ADL cilia in *osm-6(p811)* mutants by expressing *osm-6* fused to *mCherry* under control of the *sre-1* promoter was sufficient to restore wild-type *srh-234* expression in fed conditions (Figure 5C). We confirmed that ADL formed proper cilia by visually inspecting their morphology with help of *mCherry* expression, and found a significant correlation between upregulation of *srh-234* expression and wild-type cilia morphology of ADL (97% of ADL neurons with wild-type cilia expressed *srh-234p::gfp* expression at wild-type levels, n = 36). These results suggest that sensory inputs from food presence specifically through ADL cilia and dendrites are essential to promote *srh-234* expression.

We next asked whether altering the neural output of ADL neurons also effects the expression levels of *srh-234*. We therefore generated transgenic animals that express the *pkc-1(gf)* and the *tetanus toxin* (*TeTx*) cDNA under control of the ADL-specific *sre-1* promoter (*ADL::pkc-1(gf)* and *ADL::TeTx*). The *pkc-1(gf)* is a constitutively active protein kinase C that enhances synaptic output by promoting secretion of dense-core vesicles containing neuropeptides [11], [43]–[45], while *TeTx* prevents secretion of small neurotransmitter molecules by blocking synaptobrevin-mediated fusion of small, synaptic vesicles [46]. We found that expression of *ADL::pkc-1(gf)* strongly increased *srh-234* expression in fed wild-type animals compared to non-transgenic siblings (Figure 5D), while blocking ADL synaptic output by expressing *TeTx* specifically in ADL (*ADL::TeTx*) did not significantly change levels of *srh-234* expression (Figure 5D). Interestingly, the increased *srh-234* expression phenotype of *ADL::pkc-1(gf)* is completely suppressed by *npr-1(ad609)* (Figure 5D), suggesting that *npr-1* may act downstream of *pkc-1(gf)*-enhanced neuropeptide secretion from ADL to regulate *srh-234* expression. Consistent with these findings, *lf* mutations in genes that disrupt the release and processing of neuropeptides globally (e.g. *egl-3*, *unc-31* [47]–[49]), as well as an inhibitor of vesicle release (e.g. *tom-1* [50]), also modulate *srh-234* expression levels (Table S2). No changes in *srh-234* expression are observed in mutants with defects in monoamine synthesis including serotonin, octopamine and dopamine, or upon exogenous exposure to these amines (Table S2). Together, these results suggest that perhaps release of neuropeptides from ADL may in turn modulate NPR-1 in RMG to regulate *srh-234* expression.

### OCR-2 acts in ADL to regulate *srh-234* expression

We showed that intact ADL cilia are required to properly express *srh-234* in fed conditions, suggesting that a cilia-localized mechanism for sensing food presence may be important for the regulation of *srh-234*. The TRPV channels, OCR-2 and OSM-9, localize to the cilia of a subset of sensory neurons, including ADL [51], [52], and OCR-2 may function in coupling the perception of food presence to starvation survival [53]. Based on these findings, we wondered whether OCR-2 and OSM-9 may transduce sensory inputs from food into ADL to modulate *srh-234* expression. Indeed, we found that *lf* mutations in *ocr-2* (alleles *ak47* and *yz5*) strongly reduce *srh-234* expression in ADL in fed animals, whereas *osm-9(ok1667)* did so more weakly (Figure 5E; Table S2). Thus, *ocr-2* and to a lesser extent *osm-9* promote *srh-234* expression in fed conditions. It is possible that *ocr-2(ak47)* and *osm-9(ok1667)* mutants reduce *srh-234* expression when fed as a result of a decrease in food ingestion instead of a decrease in food perception; however, consistent with previous findings [54], both *ocr-2* and *osm-9* mutants ingest food similar to wild-type animals as measured by pumping rates (231.0±4 and 225±5 pumps/min for *ocr-2* and *osm-9* mutants, respectively, as compared to 235±5 pumps/min for wild-type animals, n = 25). ADL-specific expression of *ocr-2* under control of the *sre-1* promoter (*ADL::ocr-2*) fully restored *srh-234* expression in *ocr-2(ak47)* mutants during feeding, and is slightly upregulated when starved (Figure 5E); an increase likely caused by overexpression effects of the transgene. Thus, OCR-2 activity in ADL is necessary during feeding and sufficient when starved to regulate *srh-234*.

We further investigated the relationship between the OCR-2 and NPR-1 pathways in regulating *srh-234* expression. As expected, double mutants for *ocr-2(ak47); npr-1(ad609)* showed a reduced *srh-234* expression phenotype similar to that of *npr-1(ad609)* or *ocr-2(ak47)* mutants alone in both fed and starved conditions (Table S2). Interestingly, *ocr-2(ak47)* and *osm-6(p811)* fully suppressed the *unc-7(e139)* phenotype of upregulated *srh-234* expression in starved conditions (Figure 5F), suggesting that sensory inputs and OCR-2 activity are essential for the gap junction-mediated reduction of *srh-234* expression in response to starvation. Consistent with these findings, sensory inputs from inedible food that can be sensed but not eaten (Figure 2A), and ADL-specific overexpression of OCR-2 (Figure 5D), can override the effects of starvation on reducing *srh-234* expression. Thus, starvation-modulation of *srh-234* expression mediated by gap-junctions is likely dependent on sensory inputs.

### Increased calcium signaling can bypass the requirement for OCR-2, NPR-1, DAF-2 and KIN-29 in regulating the expression of *srh-234*

**Figure 6:**
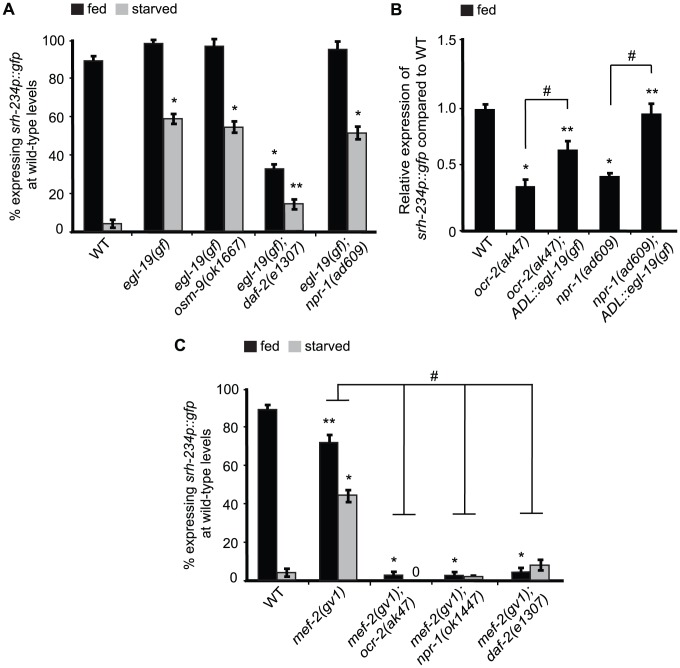
*mef-2* mutations and increased calcium signaling suppress the starvation-induced downregulation of *srh-234* expression. A, C) Percentage of animals of the indicated genotypes expressing *srh-234*p::*gfp* at wild-type levels. Animals (n>150) were examined at 150× magnification for each genotype. B) Relative expression of *srh-234*p::*gfp* in *ocr-2* and *npr-1* mutants compared to wild-type. For strains carrying *ADL::egl-19(gf)* extrachromosomal arrays (see Material and Methods), data shown is for two independent transgenic lines. Animals (n = 15–20) were examined at 400× magnification for each genotype. * and ** indicates values that are different from that of wild-type animals at *P*<0.001, and *P*<0.05, respectively, and # indicates the values that are different between the genotypes compared by brackets at *P*<0.05 using either a two-sample *t*-test or a χ^2^ test of independence. Error bars denote the SEP or SEM.

As calcium signaling plays an important role in the regulation of the KIN-29-dependent *str-1* chemoreceptor in AWB neurons [16], we explored the possibility that calcium signaling is also important for regulating *srh-234*. *gf* mutations in the voltage-gated calcium channel, *egl-19*, are predicted to prolong depolarization and result in sustained calcium influx [55]. We found that *egl-19(gf)* suppressed the starvation-induced downregulation in a wild-type background (Figure 6A), whereas *lf* mutations in *egl-19* and *unc-36*, but not *unc-2*, encoding other voltage-gated calcium channels, partially reduce *srh-234* expression during feeding (Figure S2). Thus, increased calcium signaling can override the effects of starvation on reducing *srh-234* expression. We further show that *egl-19(gf)* can suppress the reduced *srh-234* expression phenotype of *npr-1(ad609)*, *daf-2(e1307)* and *osm-9(ok1667)* mutants (Figure 6A) as well as *kin-29(oy38)* mutants (Table S2). Expression of *egl-19(gf)* specifically in ADL neurons (*ADL::egl-19(gf)*) also suppressed the reduced *srh-234* expression phenotype of *ocr-2(ak47)* and *npr-1(ad609)* mutants in fed conditions (Figure 6B). These results suggest that increased calcium signaling is sufficient in ADL to bypass the requirement of OCR-2, DAF-2 and KIN-29, and NPR-1 pathways in regulating *srh-234* expression.

To further test the hypothesis whether calcium levels in ADL may be modulated by feeding and starvation responses, we measured intracellular calcium dynamics specifically in ADL neurons in fed and starved wild-type animals in the presence of the C9-pheromone (asc-**Δ**C9; ascr#3) using the genetically encoded Ca2^+^ sensor *GCaMP3*. ADL is known to sense C9 as determined by calcium imaging and behavioral assays [12]. However, we found that C9-induced calcium transients were not significantly different in wild-type animals starved for 6 hours when compared to fed animals (Figure S3). Moreover, when we quantified the fluorescence intensity of the *ADL::GCaMP3* reporter in the absence of any stimuli in wild-type animals starved for 6, 12 or 24 hours, we also observed no significant differences in *ADL::GCaMP3* expression when compared to control fed animals (Figure S4). Thus, although we were unable to detect changes in calcium in fed and starved animals, our experiments with *egl-19(gf)* suggest that increased calcium signaling is correlated with increased *srh-234* expression.

### Mutations in *mef-2* suppress the starvation-induced downregulation of *srh-234* expression

We previously showed that *lf* mutations in *mef-2* encoding the MEF2 transcription factor can suppress the reduced *srh-234* expression phenotype of *kin-29* mutants [16], suggesting that KIN-29 antagonizes the function of MEF-2 in regulating *str-1*. We show that *mef-2(gv1)* failed to suppress the reduced *srh-234* expression phenotype of *ocr-2(ak47)*, *npr-1(ad609)* and *daf-2(e1307)* mutants in fed and starved conditions (Figure 6C), suggesting that MEF-2 does not act genetically downstream of OCR-2, DAF-2 and NPR-1. In addition, we show that *kin-29(oy38)* mutants can suppress the increased *srh-234* expression phenotype when overexpressing OCR-2 and NPR-1 (Figure 4B; Table S2), suggesting that these pathways likely act interdependently to regulate *srh-234*. Interestingly, *mef-2(gv1)* suppressed the starvation-induced downregulation of *srh-234* expression, but had no major effect on *srh-234* expression during feeding (Figure 6C), suggesting that the inhibitory regulation of *srh-234* expression by starvation signals is dependent on MEF-2 function.

Since insulin signaling appears to be compromised in *kin-29* mutants in the regulation of dauer formation and life-span [15], we next asked whether DAF-16 acts as a downstream effector of KIN-29 to regulate *srh-234* expression. However, *daf-16(mu86)* did not suppress the *srh-234* expression phenotype of *kin-29(oy38)* mutants as well as of *ocr-2(ak47)* and *npr-1(ok1447)* mutants (Table S2). Together, these results suggest that MEF-2 and DAF-16 may act as state-dependent transcriptional regulators of *srh-234* expression downstream of KIN-29 and DAF-2, respectively, while OCR-2 and NPR-1 act via MEF-2 and DAF-16-independent pathways.

## Discussion

**Figure 7:**
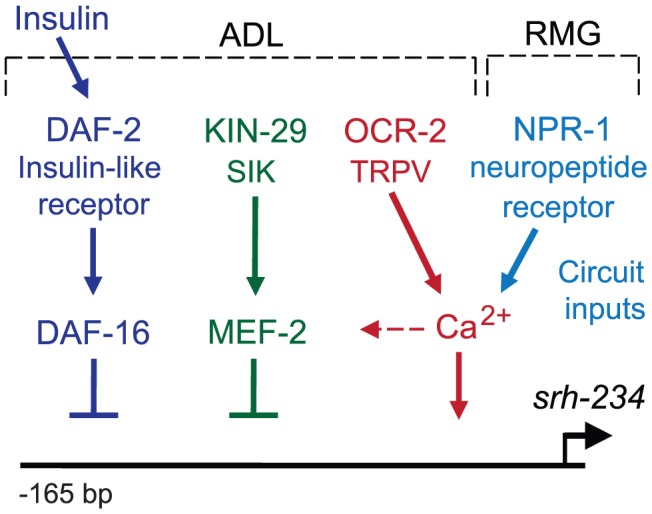
Model for sensory and circuit-mediated regulation of *srh-234* expression. Expression levels of *srh-234* are modulated by integration of sensory and internal feeding state signals via multiple pathways. During feeding, *srh-234* expression is promoted by *kin-29* salt-inducible kinase, *daf-2* insulin-like receptor, and *ocr-2* TRPV channel in ADL, and the *npr-1* neuropeptide receptor in RMG interneurons. Negative transcriptional regulators of *srh-234* expression, *mef-2* and *daf-16*, act genetically downstream of *kin-29* and *daf-2*, respectively, and likely act in parallel to *ocr-2* and *npr-1* pathways. The epistatic relationship between the different signaling pathways in ADL neurons remains to be fully defined. Unknown insulin-like peptides secreted by ADL or other neurons lead to activation of *daf-2*. Signaling mediated by *kin-29*, *ocr-2*, and *npr-1*, but less *daf-2*, converge on Ca^2+^ signaling, which likely affects activity-dependent transcription of chemoreceptor genes. After prolonged starvation, *mef-2* and *daf-16* may repress *srh-234* expression, while yet unknown transcription factors may drive *srh-234* expression during feeding.

In this study, we identified a chemoreceptor gene, *srh-234*, in the ADL sensory neuron type of *C. elegans*, whose expression levels is altered by feeding state conditions. Expression levels of *srh-234* are regulated by sensory signals associated with food presence and internal starvation signals via integration of signals by multiple pathways. In ADL neurons, signaling mediated by the *kin-29* salt-inducible kinase, the *daf-2* insulin-like receptor, and the *ocr-2* TRPV channel converge their regulation on *srh-234* expression, while the *npr-1* neuropeptide receptor acts in RMG interneurons to regulate *srh-234* expression in ADL sensory neurons (Figure 7). This sensory- and circuit-mediated regulation of chemoreceptor genes may allow animals to precisely regulate and fine-tune their chemosensory responses as a function of feeding state.

### Sensory inputs from food into ADL, and an internal state of starvation modulates chemoreceptor gene expression

Expression of *srh-234* is increased in fed animals and dramatically reduced in starved animals. When wild-type fed animals were presented with inedible *E. coli* food (bacterial food that can be sensed but not eaten after treatment with the aztreonam antibiotic), or when *eat-2(lf)* mutants were exposed to edible food, the expression of *srh-234* decreased in ADL, suggesting that the signal needed to reduce *srh-234* expression is probably an internal metabolic signal arising after food ingestion. However, when starved animals were presented with either edible or inedible *E. coli* food, expression of *srh-234* is increased in ADL, suggesting that animals perceive the availability of food, even when they are unable to eat the inedible food. Thus, inedible food likely blocks signals associated with starvation to regulate *srh-234* expression. Interestingly, this finding is similar to a previous study, showing that the repressive effects of starvation on *C. elegans* mating can be partially blocked by placing males on inedible food [24].

Sensory inputs are known to alter chemoreceptor gene expression in a subset of sensory neurons. We show that loss of all sensory inputs into ADL, as demonstrated by physically cutting the ADL dendrites, causes fed animals to reduce *srh-234* expression. Moreover, restoring only ADL cilia in *osm-6(lf)* mutants that have severe truncations of all ciliated sensory neurons [41], [42], rescues the reduced *srh-234* expression phenotype of *osm-6(lf)* mutants. These results suggest that sensory inputs through ADL sensory endings are essential to regulate *srh-234* expression. Thus, we propose that depending on food availability and feeding state, *srh-234* expression in ADL is regulated by two inputs, through external signals from food presence mediated directly by ADL and internal state signals resulting from food ingestion.

### Circuit inputs modulate chemoreceptor gene expression

Modulation of chemoreceptor gene expression is thought to be defined primarily by external environmental inputs perceived by sensory neurons and not by circuit inputs from other neurons. Our results described here reveal a novel role for circuit-mediated regulation of chemoreceptor genes in sensory neurons. First, we show that *npr-1* in RMG interneurons, which forms gap-junctions with ADL [56], was both necessary and sufficient for *srh-234* expression in ADL. Second, this *npr-1*-mediated regulation of *srh-234* is dependent on the function of *unc-7/9* gap-junctions. Third, we show that enhancing the secretion of dense-core vesicles that contain neuropeptides by expressing *pkc-1(gf)* in ADL neurons promotes expression of *srh-234*, and this regulation is dependent on *npr-1*. Fourth, we show that mutants with defects in neuropeptide processing and secretion alter the expression of *srh-234*. Lastly, insulin signals from other neurons may act on DAF-2 in ADL to regulate *srh-234* as suggested by the *srh-234* expression phenotype of *daf-28(gf)* mutants, and *daf-2/daf-16* signaling acting cell-autonomously in ADL.

The specific mechanisms by which *npr-1* in RMG interneurons alters the expression of *srh-234* in ADL remains to be determined. Current understanding suggests that NPR-1-mediated regulation of aggregative behavior and pheromone responses requires RMG [11], [12], which forms the hub of a circuit that is connected to spoke sensory neurons including ADL via gap junctions [56]. We show that *unc-7* and *unc-9* gap-junctions are essential for *npr-1*-mediated regulation of *srh-234*. Surprisingly, however, cell-specific rescue and RNAi knock down experiments of *unc-7/9* in either ADL or RMG neurons suggest that they may not be directly involved in forming gap-junctions between RMG and ADL neurons. One possibility is that *unc-7/9* are required in other neurons besides RMG and ADL for regulating *srh-234* expression, which is consistent with the partial rescue of the *srh-234* expression phenotype of *unc-7 npr-1* double mutants by driving *unc-7* cDNA in the nervous system (Figure 4D). Alternatively, any of the other innexins in *C. elegans* may act as gap-junction components within the RMG circuit.

### A role for insulin signaling in regulating chemoreceptor gene expression

Insulin signals reflect the feeding state of an animal, which has been suggested to directly affect chemosensory sensitivity to chemical cues. Our results suggest a new role for insulin-mediated regulation of chemoreceptor genes. Consistent with low *daf-2* signaling being associated with a starved state of *C. elegans*, we show that *srh-234* expression is strongly reduced in *daf-2(lf)* mutants in a *daf-16*-dependent fashion. Rescue experiments with various promoters suggest that both DAF-2 and DAF-16 act cell-autonomously in ADL to regulate *srh-234* expression. A *gf* mutation in the *daf-28* insulin-like peptide (ILP), which has been proposed to block other agonistic ILPs from binding to DAF-2 [32], reduces *srh-234* expression in fed conditions. These results indicate that ILPs from other yet unknown neurons may target DAF-2 in ADL, which in turn regulates *srh-234* expression. *C. elegans* contains 40 ILPs, and for some specific functions in particular sensory neurons have been reported. For example, in salt-chemotaxis learning, DAF-2 acts in ASE sensory neurons, and is targeted by the INS-1 ILP released from AIA interneurons [9]. Similarly, release of INS-1 from AIA regulates food-dependent AWC responses [57]. However, we show that *ins-1* mutants did not change *srh-234* expression in fed or starved conditions, suggesting there may be other ILPs involved in the regulation of *srh-234* expression.

### A role for OCR-2 and calcium in regulating chemoreceptor gene expression

The OCR-2 and OSM-9 TRPV channels in *C. elegans* have been implicated in regulating the expression of sensory neuronal genes. For example, loss of these TRPV channels reduces expression of the *odr-10* chemoreceptor gene in AWA neurons [51], [52], while they reduce the expression of the key enzyme in serotonin synthesis, *tph-1*, in AFD neurons [54]. We reveal here the first description of a chemoreceptor regulated by *ocr-2* in ADL neurons. TRPV channels in mammals are permeable to calcium [58], and in *C. elegans*, the reduced *tph-1* expression phenotype of *ocr-2(lf)* mutants can be suppressed by activating downstream calcium-dependent pathways [54]. Similarly, we show that increased calcium signaling using *egl-19(gf)* can bypass the requirement of OCR-2 in regulating the expression of *srh-234*. Such activity-dependent transcriptional regulation of chemoreceptors may allow TRPV channels to regulate the sensitivity of sensory neurons to particular chemical stimuli as a function of feeding state. MEF2 plays a critical role in activity-dependent transcription in the nervous system [59], but we show that *mef-2* does not suppress the *srh-234* reduced expression phenotype of *ocr-2* mutants. Thus, TRPV-mediated transcriptional regulation of *srh-234* is independent of MEF-2 function, suggesting other possible downstream effectors of OCR-2 in regulating *srh-234* expression. A possible target is the CREB-regulated transcriptional coactivator CRTC1, which recently was shown to regulate TRPV-mediated longevity [60], and is a known target for SIK phosphorylation [20].

### A role for MEF-2 and DAF-16 as state-dependent regulators of chemoreceptor gene expression

The specific transcriptional mechanisms by which feeding and starvation regulate chemoreceptor gene expression remain to be deciphered; however, our genetic experiments implicate a key role for the MEF-2 and DAF-16 transcription factors acting downstream of the KIN-29 and DAF-2 pathways, respectively. We show that *mef-2(lf)* and *daf-16(lf)* suppress the starvation-induced downregulation of *srh-234* expression, but the expression of *srh-234* is not altered during feeding. On the basis of these genetic experiments, we suggest that under starved conditions, MEF-2 and DAF-16 are required to repress but not activate *srh-234* expression, suggesting that other yet unknown transcription factors may be required to drive *srh-234* expression in ADL. Our previous work showed that MEF-2 is able to directly bind a MEF-2 sequence motif upstream of the *str-1* chemoreceptor [16]. Searching the upstream sequence of *srh-234* revealed a similar MEF2 binding motif as well as an E-box sequence motif (Figure S5). E-box motifs are found in the *cis*-regulatory region of genes expressed in ADL neurons [61], which are known to bind transcription factors of the basic helix-loop-helix (bHLH). Interestingly, MEF2 has been shown to interact with bHLH factors at E-boxes to regulate myogenic gene expression [62]. Thus, a similar mechanism may operate in *C. elegans*, in which MEF-2 function is essential for the starvation-induced reduction in *srh-234* expression levels by repressing a bHLH transcription factor that drives expression of *srh-234* in ADL via the E-box. The molecular mechanism by which DAF-16 regulates *srh-234* remains unclear as no canonical DAF-16 binding element (DBE) appears to be present in the 165 bp promoter sequence necessary for feeding-state regulation of *srh-234* (pers. comm. M.G and A.M.V). In *Drosophila*, SIK3 activity can be activated by insulin, which antagonizes FOXO-activated gene expression [21]. However, we show that KIN-29 antagonizes the function of MEF-2 but not DAF-16 in regulating the expression of *srh-234*, suggesting that DAF-16 mainly acts downstream of insulin signaling.

### Functional consequences of regulating chemoreceptor genes in ADL

*C. elegans* expresses multiple chemoreceptors in each chemosensory neuron. We and others have proposed that to selectively modify a response to a single chemical may rely on changing the expression of individual or subsets of chemoreceptor genes, rather than altering the response of the entire neuron, which would inadvertently result in changing the response to all stimuli sensed by that neuron. Selectively modulating distinct populations of chemoreceptors in this manner may allow *C. elegans* to modulate ‘long-term’ changes in chemosensory responses in changing environmental conditions. This expression strategy may be particularly important in ADL, which mediates avoidance responses to a wide variety of chemical signals in its environment, such as odors [63], pheromones [12], and heavy metals [64].

Our results show that the expression of least two candidate chemoreceptor genes in ADL, *srh-234* and *srh-34*, are regulated by fed and starved conditions, but it is unknown what chemical or subset of chemicals are detected by these chemoreceptors. Only a few chemoreceptors with known chemical ligands have been found in *C. elegans*, such as the chemoreceptor gene, *odr-10*, expressed in the AWA olfactory neuron type for the attractive chemical diacetyl [65]. When the *odr-10* gene was introduced into the repulsive AWB neuron, *C. elegans* changes its normally attractive response to diacetyl to trigger avoidance of diacetyl [66], suggesting that chemoreceptors expressed in a specific neuron type are linked to a common odor response that is determined by the identity of the neuron. Similarly, all chemoreceptors expressed in the ADL neuron type may be linked to a common avoidance response to chemicals detected by ADL, although in some cases a sensory neuron has the capacity to switch its behavioral preference towards odors [45]. The presence and absence of food is known to rapidly and reversibly alter responses of animals to repulsive chemical cues, and part of this response appears to be mediated by ADL [7]. It is therefore tempting to speculate that increased *srh-234* expression in ADL, allows fed animals to be less tolerant of aversive stimuli detected by *srh-234*, whereas decreased *srh-234* expression in ADL following starvation may allow starved animals to be more tolerant to these aversive stimuli. This dynamic modulation in chemoreceptor gene expression could allow starved *C. elegans* animals to sample different environments, perhaps increasing their chances of finding food under stress-full conditions.

### Plasticity in chemoreceptor gene expression in other systems

Other invertebrates and vertebrates alter chemoreceptor gene expression in response to environmental and developmental signals. For example, expression of olfactory receptor genes in the zebrafish *Danio rerio* is induced in temporal waves, which may reflect a mechanism for odorant sensitivity during development [67]. Changes in chemoreceptor gene expression is also dependent on the sex of an animal. After mating, female flies of *Drosophila* rapidly modify their chemosensory behaviors to lower their attraction to males such that they can focus on reproduction, and these behavioral changes are accompanied by modulation of different chemoreceptor genes [68]. Interestingly, in mosquitoes, the regulated expression of a subset of olfactory receptor genes before and after a blood feeding has been correlated with transitions between host-seeking and leaving behavior [2]–[5]. Parasitic nematodes also actively seek out their host using chemical cues (reviewed in [69]); however, little is known about host-seeking behaviors in parasitic nematodes, and even less is known about whether expression of chemoreceptor genes are modulated by its feeding state. The function of certain chemosensory neuron types in parasitic nematodes have been examined in only a few cases. For example, similar to *C. elegans*, ablation of ASE and ASH neurons of the parasitic nematode *S. stercoralis* showed that these sensory neuron types mediate attraction and repulsion to soluble chemicals, respectively [70]. Given that *C. elegans* has high anatomical and functional similarity to certain parasitic nematodes, it would be important to determine whether similar mechanisms of feeding state-regulated chemoreceptor gene expression presented here operate in parasitic nematodes. Moreover, identification of additional regulators and mechanisms underlying chemoreceptor gene expression will lead to a better understanding of how animals modify their chemosensory behavior in response to changes in external and internal conditions.

## Materials and Methods

### Strains

Nematodes were grown at 20°C under standard conditions [71] on nematode growth medium (NGM) with *E. coli* OP50 as the primary food source unless indicated otherwise. The wild-type strain used was *C. elegans* variety Bristol, strain N2. Mutant strains used in this study were obtained from the *Caenorhabditis* Genetics Center (CGC) unless indicated otherwise: PR811 *osm-6(p811)*, PR813 *osm-5(p813)*, DA609 *npr-1(ad609)*, RB1330 *npr-1(ok1447)*, CX4148 *npr-1(ky13)*; CX4057 *npr-1(g320)*; VC2106 *flp-18(gk3063)*, RB982 *flp-21(ok889)*, VC461 *egl-3(gk328)*, CB169 *unc-31(e169)*, CB55 *unc-2(e55)*, CB251 *unc-36(e251)*, CB1370 *daf-2(e1370)*, JT191 *daf-28(sa191)*, CF1038 *daf-16(mu86)*, LC33 *bas-1(tm351)*, CB1112 *cat-2(e1112)*, RB1161 *tbh-1(ok1196)*, RB993 *tdc-1(ok914)*, GR1321 *tph-1(mg280)*, VC1262 *osm-9(ok1677)*, CX4544 *ocr-2(ak47)*, JY243 *ocr-2(yz5)*, DA465 *eat-2(ad465)*, DA1116 *eat-2(ad1116)*, KM134 *mef-2(gv1)*, PY1476 *kin-29(oy38)*, CB139 *unc-7(e139)*, CB101 *unc-9(e101)*, MT6129 *egl-19(n2368)gf*, MT1212 *egl-19(n582)*, VC223 *tom-1(ok285)*, and DR476 *daf-22(m130)*. Transgenic strains used in this study were: VDL3 *oyIs56*[*srh-234*p::*gfp, unc-122*p::*rfp*], VDL143 *oyIs57*[*srh-234*p::*gfp, unc-122*p::*rfp*], and BOL171 *npr-1(ad609); lin-15*[*ncs-1*p::*Cre flp-21*p::*loxPstoploxP::npr-1 SL2 gfp, lin-15(+)*] (*RMG::npr-1*) [11], and *sre-1::GCaMP3* [12]. Double mutant strains were constructed using standard genetic methods, and the presence of each mutation was confirmed via PCR or sequencing.

### Real-time qRT PCR

Total RNA was isolated from a growth-synchronized population of adult animals and reverse transcribed using oligo(dT) primers. Real-time quantitative reverse transcription–PCR (qRT–PCR) was performed with a Corbett Research Rotor-Gene 3000 real-time cycler, Platinum Taq polymerase (Invitrogen), and primers specific for *srh-234* and *odr-10* coding sequences. Primer sequences for *srh-234* are 5′-GGACAATTGAAATGCAACACA-3′ and 5′-GACGGGGACAATAAAGAGCA-3′. Primer sequences for *odr-10* are 5′-GAGAATTGTGGATTACCCTAG-3′ and 5′-CTCAATATGCATTATAGGTCGTAATATG-3′.

### Measurement and quantification of *srh-234*p::*gfp*

Animals carrying *srh-234*p::*gfp* reporters were cultured and grown at 20°C on standard nematode growth media (NGM) plates seeded with *E. coli* OP50 as the bacterial food source unless indicated otherwise. Young adult animals were washed with M9 buffer (to remove any bacteria in the gut) and transferred onto plates with *E. coli* OP50 food (Fed) or without *E. coli* OP50 food for 12–24 hours (Starved) unless indicated otherwise. Levels of *srh-234*p::*gfp* expression in animals were measured under a dissection microscope equipped with epifluorescence as described [16]. For quantification, *gfp* was scored as “bright” if levels of *gfp* fluorescence allowed visualization of one of the ADL cell bodies and dendritic process, and “dim” if *gfp* expression could not be detected or could be detected weakly at the same magnification. For more precise measurements, we mounted animals on a DM5500 compound microscope, and used Volocity analytical software to quantify *gfp* expression levels emanating from the *srh-234*p::*gfp* reporter. Statistical analyses of *srh-234* expression were performed using either the two-sample *t*-test, or the χ^2^ test of independence to test for statistically significant differences between proportions in the categories “bright” and “dim” for different genotypes (d.f. 1). A proportion of 0% was set to a default of 1.

### Analysis of *srh-234*p::*gfp* expression

Different food conditions: We exposed fed and starved animals carrying *srh-234*p::*gfp* reporters to aztreonam-treated *E. coli* OP50 (inedible food). For generating inedible food, *E. coli* OP50 was treated with the aztreonam antibiotic as previously described [24]. In brief, *E. coli* OP50 was grown in LB to log phase at 37°C with shaking. Cultures were mixed with the aztreonam antibiotic (Sigma) to a final concentration of 10 µg/ml and incubated for an additional 3 hours at 37°C with minimal shaking to prevent bacterial shearing. Bacteria was spread onto NGM plates containing 10 µg/ml aztreonam and immediately dried and used the same day, since the septum inhibitory effects of aztreonam are short lived. Expression levels of *srh-234*p::*gfp* were measured and quantified following exposure to the different food conditions as described above.

Sephadex-beads: We exposed starved wild-type animals carrying *srh-234*p::*gfp* reporters to 1 ml of 30 mg/ml Illustra Sephadex G-50 DNA Grade Fine (GE Healthcare UK Limited) suspended in water spread on NGM agar plates without bacterial food as described previously [72]. After at least 6 hours of exposure to Sephadex beads, animals were rapidly transferred into 35% sucrose solution to separate animals from the Sephadex beads. Animals floating on the surface of the sucrose solution were collected in 1× M9 buffer and immediately transferred to NGM agar plates without food for measurement and quantification of *srh-234*p::*gfp* expression levels. We confirmed that the sucrose floating procedure alone does not alter *srh-234*p::*gfp* expression levels.

Monogenic amines: We exposed fed or starved wild-type animals carrying *srh-234*p::*gfp* reporters to NGM plates with or without 3 mg/ml octopamine-hydrochloride (Sigma) in the presence of food, and 1 mg/ml serotonin creatinine sulphate (Sigma) in the absence of food. For octopamine treatment, an overnight culture of *E. coli* OP50 in LB was spun down and resuspended in 1/20 volume of water. About 50 µl of the concentrated OP50 was spread on the assay plates and was left until the surface of the plates became dry. After 6 hours of exposure to serotonin and octopamine, *srh-234*p::*gfp* expression levels were measured and quantified.

Pheromones: We exposed fed wild-type animals carrying *srh-234*p::*gfp* reporters to low levels of crude dauer pheromone in the presence of UV-killed *E. coli* food as described previously [13]. Dauer pheromone was prepared according to Golden and Riddle [22].

### Expression constructs and generation of transgenic animals

Expression vectors were generated by amplifying either the wild-type genomic sequences of *osm-6*, *npr-1*, *ocr-2*, *egl-19(gf)*, or cDNAs of *daf-2* ([73], a kind gift from Shreekanth Chalasani), *daf-16a* (this study), *unc-7*, *unc-9* (this study), *pkc-1(gf)* and *tetanus toxin* (*TeTx*) (kind gift from Cori Bargmann, [12]). This resulted in the generation of the constructs pMG1 *sre-1p::osm-6 genomic::mCherry* (*ADL::osm-6*), pMG2 *sre-1p::npr-1* genomic::*mCherry* (*ADL::npr-1*), pMG3 *sre-1p::ocr-2* genomic::*mCherry* (*ADL::ocr-2*), pMG4 *sre-1::pkc-1(gf)* cDNA (*ADL::pkc-1(gf)*), pMG5 *sre-1p::tetanus toxin* cDNA (*ADL::TeTx*), pVDL14 *sre-1p::daf-2* cDNA *SL2::mCherry* (*ADL::daf-2*), pMG14 *sre-1p::daf-16a* cDNA *SL2::mCherry* (*ADL::daf-16a*), pMG24 *sre-1p::unc-7* cDNA *SL2::mCherry* (*ADL::unc-7*), and pMG39 *flp-21::unc-7* cDNA *SL2::mCherry* (*flp-21::unc-7*), pMG40 *unc-31p::unc-7* cDNA *SL2::mCherry* (*pan-neural::unc-7*), and pMG37 *sre-1p::egl-19(gf)* genomic *SL2::mCherry* (*ADL::egl-19(gf)*). Expression constructs *rgef-1p::daf-2* (pJH664) (*pan-neural::daf-2*), *ges-1p::daf-2* (pJH668) (*intestine:daf-2*), and *ges-1p::gfp::daf-16a* (pJH2973) (*intestine::daf-16a*) are kind gifts from Mei Zhen [32]. For generating the *npr-1*p::*npr-1* expression construct, we fused 2.5 Kb of the *npr-1* regulatory sequence and the complete *npr-1* wild-type genomic sequence to *gfp* as previously described [74]. Transgenic animals were generated using the *unc-122*p::*dsRed* (50–100 ng/µl) or the pRF4 *rol-6(su1006)* co-injection markers injected at 150 ng/µl. Expression constructs were injected at 20 ng/µl. For generating the cell-specific knock down constructs, we fused the *sre-1* promoter to a 1 kb exon-rich genomic fragment of either *unc-7* or *unc-9* in the sense and anti-sense (sas) orientation as described [75]. Equal amounts of PCR products of either *ADL::unc-7(sas)* or *ADL::unc-9(sas)* were mixed and microinjected together with the pRF4 *rol-6(su1006)* co-injection markers injected at 90 ng/µl. All amplified products in the generated constructs were sequenced to confirm the absence of errors generated via the amplification procedure.

### Subcellular laser ablation

Laser microsurgery was carried out as previously described [39]. A titanium::sapphire laser system (Mantis PulseSwitch Laser, Coherent Inc., Santa Clara, CA) generated a 1 kHz train of near infrared (λ = 800 nm) pulses that were ∼100 fs in duration and had a pulse energy of 5–15 nJ. The laser beam was focused to a diffraction-limited spot (using a 60× microscope objective) and used to disrupt sensory dendrites of ADL neurons expressing *srh-234*p::*gfp*. Following brief laser exposure, the targeted dendrite was inspected for a visual break to confirm that is was severed. Prior to laser surgery of either the ADLL or ADLR sensory dendrite, fed animals were aestheticized on 2% agar pads with 3 mM Sodium Azide, removed post-surgery for recovery and returned to NGM agar plates containing *E. coli* OP50 for measuring and quantifying *srh-234*p::*gfp* expression levels 2, 4, 6 and 24 hours following surgery in fed animals. Expression of *srh-234* in severed (“cut”) neurons was compared to controls (“un-cut”) neurons in the same animals that were aestheticized for imaging but received no laser surgery.

### Quantification of *sre-1*p::*GCaMP3* in fed and starved animals

To measure fluorescence intensity of *GCaMP3* expressed specifically in ADL neurons under either fed or starved conditions, three L4 staged *sre-1*p::*GCaMP3* integrated transgenic animals [12] were grown on a *E. coli* OP50 seeded plate for 1.5 days to obtain about 100 eggs. The eggs were grown until they became young adults at 20°C. The young adult animals were washed in 1× M9 buffer and divided and placed on two NGM plates (one plate in the presence of *E. coli* OP50 food: “fed”, and one plate in the absence of *E. coli* OP50 food, “starved”). Images of *GCaMP3* expression in the ADL neurons were captured under fixed exposure time (500 ms) with a Zeiss Axioplan microscopy using a 40× objective and Zeiss AxioCam HR camera at 0 hr, 6 hr, 12 hr or 24 hr in either fed or starved conditions (n = 40–60 for each). The fluorescence intensity of *GCaMP3* in ADL was quantified using the Image J software (NIH).

### Imaging of C9 ascaroside-induced Ca^2+^ responses in fed and starved animals

Ca^2+^ imaging experiments in the presence of C9 (asc-**Δ**C9; ascr#3) pheromone were carried out as previously described [12] with custom-made microfluidics chips [76]. Imaging was performed on a Zeiss Axioplan microscopy using a 40× objective and a Zeiss AxioCam HR camera. The images were analyzed using Image J software (NIH), and a custom-written MATLAB (The Mathworks) script.

## Supporting Information

Figure S1 Re-feeding restores *srh-234* expression in starved animals. A) Percentage of starved wild-type L1 larvae (red line), and adults (black line) expressing *srh-234*p::*gfp* at wild-type levels at different time-points (hr) after they were placed on-food (*E. coli* OP50) plates. B) Percentage of starved animals expressing *srh-234*p::*gfp* at wild-type levels at different time-points (hr) after they were placed on-food (*E. coli* OP50) plates (black line), off-food plates (red line), or axenic-media without food (blue line). Error bars denote the SEP.
(EPS)

Figure S2 ADL-specific RNAi of *unc-7* and *unc-9* does not suppress the *npr-1*-mediated reduction of *srh-234* expression. Relative expression of *srh-234*p::*gfp* in the indicated genotypes compared to wild-type animals. For strains carrying *ADL::unc-7(sas)* and *ADL::unc-9(sas)* extrachromosomal arrays, data shown is for at least two independent transgenic lines. Animals (n = 10–25) were examined at 400× magnification for each genotype. * indicates values that are different from that of wild-type animals at *P*<0.001 using a two-sample *t*-test. n.s. indicates values that are not significantly different. (sas) indicates sense-antisense. Error bars denote the SEM.
(EPS)

Figure S3 C9 pheromone-induced Ca2^+^ transients in the ADL neurons of well-fed or starved wild-type animals. (Left) Intracellular Ca2^+^ dynamics in *GCaMP3*-expressing ADL neurons upon addition of 500 nM C9 (asc-**Δ**C9; ascr#3) (red horizontal bars) were observed in animals at 0 hr (A) or 6 hr (B) after they were placed on-food or off-food plates, respectively. (Middle) Scatter plot shows the peak percentage changes after C9 addition. Dotted lines indicate the median. (Right) The averages of the peak percentage change after C9 addition are shown. n≥10 neurons each. n.s. indicates the values between brackets that are not significantly different. Error bars denote the SEM.
(EPS)

Figure S4 Fluorescence intensity of the genetically encoded Ca2^+^ sensor *GCaMP3* specifically expressed in ADL of fed or starved wild-type animals. Fluorescence intensity (A.U.) was measure at 0, 6, 12 and 24 hr after they were placed on-food or off-food plates. # indicates the values that are different between the brackets at *P*<0.05 using a two-sample *t*-test. n≥40 animals for each. Error bars denote the SEM.
(EPS)

Figure S5 The promoter sequence of *srh-234* contains a putative MEF-2 and E-box sequence motif. Expression of *gfp* driven by 165 bp *srh-234* regulatory sequence. Shown is a predicted E-box motif (stippled box) that drives expression of ADL-expressed genes [61], and a predicted MEF2 site (black box).
(EPS)

Table S1 Summary of ADL-expressed chemoreceptor genes examined as a function of feeding state. ^a^ Expression of *gfp* gene fusions carried on extrachromosomal arrays, and arrays stably integrated into the genome, *gmIs12*[*srb-6*p::*gfp*] and *oyIs56*[*srh-234*p::*gfp*] were examined in adult animals in fed and starved conditions. ^b^ “+”, regulated, or “−” not regulated by fed and starved conditions. n = 150–250.
(DOCX)

Table S2 Analysis of *srh-234p::gfp* expression levels as a function of feeding state in different conditions and mutants. ^a^ Adult animals grown at 20°C in the presence of OP50 food were examined in all cases unless indicated otherwise. All strains contain stably integrated copies of *oyIs56*[*srh-234*p::*gfp*] fusion genes with the exception of *bas-1* and *unc-36* which contain integrated copies of *oyIs57*[*srh-234p::gfp*]. ^b^ Expression of *oyIs56*[*srh-234*p::*gfp*] was examined at 150× magnification as defined in Material and Methods. ^c^ Expression of *oyIs57*[*srh-234*p::*gfp*] was examined at 400× magnification as defined in Material and Methods. ^d^ Indicates values that are different from that of wild-type animals either in fed or starved conditions using a χ^2^ test of independence. ^e^ Compared to wild-type *oyIs56[srh-234p::gfp* when fed. ^f^ Compared to wild-type *oyIs57[srh-234p::gfp]* when fed. ^g^ Compared to wild-type *oyIs56[srh-234p::gfp* when starved. ^h^ Compared to *kin-29(oy38)* under same conditions. ^i^ Compared to *unc-9(e101)* under same conditions. ^j^ Compared to *unc-7(e139)* under same conditions. n = 150–350.
(DOCX)
