# Supplementary material for: Feeding State, Insulin and NPR-1 Modulate Chemoreceptor Gene Expression via Integration of Sensory and Circuit Inputs
Source: PLoS Genet. 2014 Oct 30;10(10):e1004707. doi: 10.1371/journal.pgen.1004707 (PMC4214617; doi:10.1371/journal.pgen.1004707)
Supplement: Table S1 — Summary of ADL-expressed chemoreceptor genes examined as a function of feeding state. a Expression of gfp gene fusions carried on extrachromosomal arrays, and arrays stably integrated into the genome, gmIs12[srb-6p::gfp] and oyIs56[srh-234p::gfp] were examined in adult animals in fed and starved conditions. b “+”, regulated, or “−” not regulated by fed and starved conditions. n = 150–250. (DOCX) [file pgen.1004707.s006.docx]

**Table S1.** Summary of ADL-expressed chemoreceptor genes examined as a function of feeding state.

| Gene^a^ | Feeding state  regulated^b^ |
| --- | --- |
| *srb-6*  *sre-1*  *srh-34*  *srh-37*  *srh-60*  *srh-132*  *srh-186*  *srh-220*  *srh-234*  *sri-51*  *sro-1*  *srz-24*  *srz-78* | _  _  +  _  _  _  _  _  +  _  _  _  _ |

n = 150-250

^a^ Expression of *gfp* gene fusions carried on extrachromosomal arrays, and arrays stably integrated into the genome, *gmIs12*[*srb-6*p*::gfp*] and *oyIs56*[*srh-234*p*::gfp*] were examined in adult animals in fed and starved conditions.

^b^ “+”, regulated, or “-“ not regulated by fed and starved conditions.
