# Supplementary material for: Feeding State, Insulin and NPR-1 Modulate Chemoreceptor Gene Expression via Integration of Sensory and Circuit Inputs
Source: PLoS Genet. 2014 Oct 30;10(10):e1004707. doi: 10.1371/journal.pgen.1004707 (PMC4214617; doi:10.1371/journal.pgen.1004707)
Supplement: Table S2 — Analysis of srh-234p::gfp expression levels as a function of feeding state in different conditions and mutants. a Adult animals grown at 20°C in the presence of OP50 food were examined in all cases unless indicated otherwise. All strains contain stably integrated copies of oyIs56[srh-234p::gfp] fusion genes with the exception of bas-1 and unc-36 which contain integrated copies of oyIs57[srh-234p::gfp]. b Expression of oyIs56[srh-234p::gfp] was examined at 150× magnification as defined in Material and Methods. c Expression of oyIs57[srh-234p::gfp] was examined at 400× magnification as defined in Material and Methods. d Indicates values that are different from that of wild-type animals either in fed or starved conditions using a χ2 test of independence. e Compared to wild-type oyIs56[srh-234p::gfp when fed. f Compared to wild-type oyIs57[srh-234p::gfp] when fed. g Compared to wild-type oyIs56[srh-234p::gfp when starved. h Compared to kin-29(oy38) under same conditions. i Compared to unc-9(e101) under same conditions. j Compared to unc-7(e139) under same conditions. n = 150–350. (DOCX) [file pgen.1004707.s007.docx]

**Table S2.** Analysis of *srh-234p::gfp* expression levels as a function of feeding state in different conditions and mutants.

| Strain^a^ | Feeding  State | % expressing  *srh-234p::gfp* in at least one ADL neuron^b^ | *P*- values^d^ |
| --- | --- | --- | --- |
| *oyIs56[srh-234p::gfp]*  *oyIs56[srh-234p::gfp]*  *oyIs57[srh-234p::gfp]^c^*  *oyIs57[srh-234p::gfp]^c^*  Bacterial food  HB101  HB101  Pheromone  *daf-22(m130)*  *daf-22(m130)*  Crude pheromone + OP50 food  Monoamine synthesis  *tdc-1(ok914)*  *tdc-1(ok914)*  *tbh-1(ok1196)*  *tbh-1(ok1196)*  *tph-1(mg280)*  *tph-1(mg280)*  *cat-2(e1112)*  *cat-2(e1112)*  *bas-1(tm315)^c^*  *bas-1(tm315)^c^*  Exogenous Amines and Sephadex  Serotonin + OP50 food  Octopamine + OP50 food  Sephadex beads + no food  Double mutants with *kin-*29  *kin-29(oy38)*  *kin-29(oy38); mef-2(gv1)*  *kin-29(oy38); egl-19(gf)*  *kin-29(oy38); daf-16(mu86)*  *kin-29(oy38) npr-1(ad609)*  *kin-29(oy38) unc-7(e139)*  *kin-29(oy38); Ex*[*ADL::ocr-2*]  *kin-29(oy38); Ex*[*npr-1::npr-1*]  Cilia defective  *osm-5(p813)*  Insulin signaling  *ins-1(tm1888)*  *ins-1(tm1888)*  TRPV signaling  *ocr-2(yz5)*  *ocr-2(yz5)*  Double mutants with *ocr-2*  *ocr-2(ak47)*  *ocr-2(ak47)*  *ocr-2(ak47); npr-1(ad609)*  *ocr-2(ak47); npr-1(ad609)*  *ocr-2(ak47); daf-16(mu86)*  *ocr-2(ak47); daf-16(mu86)*  NPR-1 signaling  *npr-1(g320)*  *npr-1(g320)*  *npr-1(ok1447)*  *npr-1(ok1447)*  *npr-1(ky13)*  *npr-1(ky13)*  *flp-18(gk3063)*  *flp-18(gk3063)*  *flp-21(ok889)*  *flp-21(ok889) flp-18(gk3063); flp-21(ok889)*  *flp-18(gk3063); flp-21(ok889*  *npr-1(ok1447); daf-16(mu86)*  *npr-1(ok1447); daf-16(mu86)*  Double mutants with *unc-9*  *unc-9(e101)*  *unc-9(e101)*  *unc-9(e101) npr-1(ad609)*  *unc-9(e101) npr-1(ad609)*  *unc-9(e101); osm-6(p811)*  *unc-9(e101); osm-6(p811)*  *unc-9(e101); ocr-2(ak47)*  *unc-9(e101); ocr-2(ak47)*  Double mutants with *unc-7*  *unc-7(e139)*  *unc-7(e139)*  *unc-7(e139); npr-1(ky13)*  *unc-7(e139); npr-1(ky13)*  *unc-7(e139); daf-2(e1307)*  *unc-7(e139); daf-2(e1307)*  Neuropeptide release and processing  *unc-31(e169)*  *unc-31(e169)*  *tom-1(ok285)*  *tom-1(ok285)*  *egl-3(gk328)*  *egl-3(gk328)*  Voltage-gated calcium channels  *unc-2(e55)*  *unc-2(e55)*  *egl-19(n582)*  *egl-19(n582)*  *unc-36(e251)^c^*  *unc-36(e251)^c^* | Fed  Starved  Fed  Starved  Fed  Starved  Fed  Starved  Fed  Fed  Starved  Fed  Starved  Fed  Starved  Fed  Starved  Fed  Starved  Starved  Fed  Starved  Fed  Fed  Fed  Fed  Fed  Fed  Fed  Fed  Fed  Fed  Starved  Fed  Starved  Fed  Starved  Fed  Starved  Fed  Starved  Fed  Starved  Fed  Starved  Fed  Starved  Fed  Starved  Fed  Starved  Fed  Starved  Fed  Starved  Fed  Starved  Fed  Starved  Fed  Starved  Fed  Starved  Fed  Starved  Fed  Starved  Fed  Starved  Fed  Starved  Fed  Starved  Fed  Starved  Fed  Starved  Fed  Starved  Fed  Starved | 87  5  89  6  88  6  98  10  88  80  0  87  2  91  5  82  4  98  1  8  98  2  0  91  67  0  0  0  0  0  0  93  2  0  0  2  1  1  1  1  1  52  2  6  2  17  1  92  18  80  4  94  2  0  0  96  95  95  93  0  0  0  0  97  96  84  16  2  0  100  22  35  1  96  47  96  5  60  0  38  0 | <0.001^e^  <0.001^f^  <0.001^h^  <0.001^h^  <0.001^e^  <0.001^e^  <0.001^e^  <0.001^e^  <0.001^e^  <0.001^e^  <0.05^g^  <0.001^g^  <0.001^g^  <0.001^i^  <0.001^i^  <0.001^g^  <0.05^g^  <0.001^j^  <0.05^g^  <0.001^e^  <0.001^g^  <0.001^e^  <0.001^f^ |

n = 150-350.

^a^ Adult animals grown at 20°C in the presence of OP50 food were examined in all cases unless indicated otherwise. All strains contain stably integrated copies of *oyIs56*[*srh-234*p*::gfp*] fusion genes with the exception of *bas-1* and *unc-36* which contain integrated copies of *oyIs57*[*srh-234p::gfp*].

^b^ Expression of *oyIs56*[*srh-234*p*::gfp*] was examined at 150X magnification as defined in Material and Methods.

^c^ Expression of *oyIs57*[*srh-234*p*::gfp*] was examined at 400X magnification as defined in Material and Methods.

^d^ Indicates values that are different from that of wild-type animals either in fed or starved conditions using a χ^2^ test of independence.

^e^ Compared to wild-type *oyIs56[srh-234p::gfp* when fed.

^f^ Compared to wild-type *oyIs57[srh-234p::gfp]* when fed.

^g^ Compared to wild-type *oyIs56[srh-234p::gfp* when starved.

^h^ Compared to *kin-29(oy38)* under same conditions.

^i^ Compared to *unc-9(e101)* under same conditions.

^j^ Compared to *unc-7(e139)* under same conditions.
